# Supplementary material for: Generation of functional liver organoids on combining hepatocytes and cholangiocytes with hepatobiliary connections ex vivo
Source: Nat Commun. 2021 Jun 7;12:3390. doi: 10.1038/s41467-021-23575-1 (PMC8185093; doi:10.1038/s41467-021-23575-1)
Supplement: Supplementary file 1 — Supplementary Information [file 41467_2021_23575_MOESM1_ESM.pdf]

SUPPLEMENTARY INFORMATION

Generation of functional liver tissue on combining hepatocytes and cholangiocytes with hepatobiliary connections *ex vivo*

Tanimizu N. et al.

Supplementary Tables 1-4

Supplementary Figures 1-21

Supplementary Table 1. Lineage of hepatocytes and cholangiocytes in HBTO

	Cell Count /Area (cells)		Ratio (%)	
Wt SH & tdTomato$^+$ Cholangiocyte	tdTomato$^+$HNF4α$^-$	tdTomato$^+$HNF4α^{low}	tdTomato$^+$HNF4α$^-$	tdTomato$^+$HNF4α^{low}
	99.0±15.3	0.5±0.5	99.3±0.6	0.7±0.6
tdTomato$^+$ SH & Wt Cholangiocyte	tdTomato$^+$CK19$^-$	tdTomato$^+$CK19$^+$	tdTomato$^+$CK19$^-$	tdTomato$^+$CK19$^+$
	46.8±9.8	0	100	0

In co-culture of wild type (Wt) SHs and tdTomato$^+$ cholangiocytes, some tdTomato$^+$HNF4α$^+$ cells were observed, suggesting that cholangiocyte-to-hepatocyte conversion proceeds in HBTOs. However, HNF4α expression in those cells was quite low, and they maintained the cellular morphology of cholangiocytes. In co-cultures of tdTomato$^+$ SHs and Wt cholangiocytes, tdTomato$^+$CK19$^+$ cells were not observed.

Supplementary Table 2. Primary Antibodies

Antibody	Company	Host animal	Method	Dilution
Albumin	Bethyl laboratory	goat	IF	1:1000
CD16/32	BD Pharmingen	rat	FACS	1:1000
CD31 (PE-Cy7-conjugated)	BD Pharmingen	rat	FACS	1:1000
CD45 (APC-Cy7-conjugated)	BD Pharmingen	rat	FACS	1:1000
CEACAM	R&D systems	sheep	IF	1:500
CLDN2	Proteintech	rabbit	IF	1:500
CYP3A4	Abcam	rabbit	IF	1:400
Cytokeratin 19	Tanimizu et al. 2003	rabbit	IF	1:2000
ECAD	BD biosciences	mouse	IF	1:1000
ECAD (PE-conjugated)	Biolegend	rat	FACS	1:1000
EpCAM	BD Pharmingen	rat	IF	1:500
EpCAM (FITC-conjugated)	Biolegend	rat	MACS	1:1000
EZRIN	Proteintech	rabbit	IF	1:500
HNF4□	SantaCruz Biotechnology Inc.	rabbit	IF	1:200
HNF4□	SantaCruz Biotechnology Inc.	goat	IF	1:200
ICAM-1 (PE-conjugated)	BD Pharmingen	rat	FACS	1:1000
OPN	R&D systems	goat	IF	1:500
RADIXN	Abcam	rabbit	IF	1:500
SOX9	Millipore	rabbit	IF	1:1000
ZO1	A gift from Dr. Bruce Stevenson (University of Alberta)	rat	IF	1:2000

Supplementary Table 3. Secondary Antibodies

Antibody	Company	Host animal	Method	Dilution
AlexaFluor488 anti-rabbit IgG	Thermofisher Scientific	Donkey	IF	1:1000
AlexaFluor555 anti-rabbit IgG	Thermofisher Scientific	Donkey	IF	1:1000
AlexaFluor647 anti-rabbit IgG	Thermofisher Scientific	Donkey	IF	1:1000
AlexaFluor488 anti-goat IgG	Thermofisher Scientific	Donkey	IF	1:1000
AlexaFluor555 anti-goat IgG	Thermofisher Scientific	Donkey	IF	1:1000
AlexaFluor633 anti-goat IgG	Thermofisher Scientific	Donkey	IF	1:1000
AlexaFluor488 anti-sheep IgG	Thermofisher Scientific	Donkey	IF	1:1000
Cy3 anti-sheep IgG	Jackson Immuno Research Laboratories Inc.	Donkey	IF	1:1000
AlexaFluor488 anti-rat IgG	Thermofisher Scientific	Donkey	IF	1:1000
Cy5 anti-rat IgG	Jackson Immuno Research Laboratories Inc	Donkey	IF	1:1000
AlexaFluor488 Phalloidin	Thermofisher Scientific	Donkey	IF	1:300
AlexaFluor555 Phalloidin	Thermofisher Scientific	Donkey	IF	1:300
AlexaFluor633 Phalloidin	Thermofisher Scientific	Donkey	IF	1:300

Supplementary Table 4. Primers used for PCR

Gene name		Sequence
Ae2	Sense	5′-CAGCAAAGGGGCACAGAC-3′
	Antisense	5′-CACCTCCGTCGTCACCTC-3′
Albumin	Sense	5′-GAA AGC CCA CTG TCT TAG TG-3′
	Antisense	5′-GGG TGT AGC GAA CTA GAA TG-3′
Axin2	Sense	5′-GAGAGTGAGCGGCAGAGC-3′
	Antisense	5′-CGGCTGACTCGTTCTCCT-3′
Cdh1	Sense	5′-ATCCTCGCCCTGCTGATT-3′
	Antisense	5′-ACCACCGTTCTCCTCCGTA-3′
Cftr	Sense	5′-ATGTGGCCTCACTGTCCTTC-3′
	Antisense	5′-GCTGATCGAGGTCTAAGAGCA-3′
Cldn2	Sense	5′-TGTGAATGAACTGAAGGAAAGC-3′
	Antisense	5′-ATCCTGCACCCAGCTGTATT-3′
Cyp1a2	Sense	5′-CCCTGCCCTTCAGTGGTACA-3′
	Antisense	5′-AAGCTGTAGAGGTCTGGTCG-3′
Cyp2e1	Sense	5′-GGAACACCTTAAGTCACTGGACA-3′
	Antisense	5′-ATGGGTTCTTGGCTGTGTTT-3′
Cyp3a11	Sense	5′-TGAATATGAAACTTGCTCTCACTAAAA-3′
	Antisense	5′-CCTTGTCTGCTTAATTTCAGAGGT-3′
Cyp7a1	Sense	5′-TCAAGCAAACACCATTCCTG-3′
	Antisense	5′-GGCTGCTTTCATTGCTTCA-3′
Glu1	Sense	5′-CTCGCTCTCCTGACCTGTTC-3′
	Antisense	5′-TTCAAGTGGGAACTTGCTGA-3′
Gapdh	Sense	5′-ACC ACA GTC CAT GCC ATC AC-3′
	Antisense	5′-TCC ACC ACC CTG TTG CTG TA-3′
Lgr5	Sense	5′-CTTCACTCGGTGCAGTGCT-3′
	Antisense	5′-CAGCCAGCTACCAAATAGGTG-3′
Tdo2	Sense	5′-TGAGTAAAGGTGAACGACGAC-3′
	Antisense	5′-AGCCGACTGAGAATCCTGTA-3′
tdTomato	Sense	5′-CTGTTCCTGTACGGCATGG-3′
	Antisense	5′-GGCATTAAAGCAGCGTATCC-3′

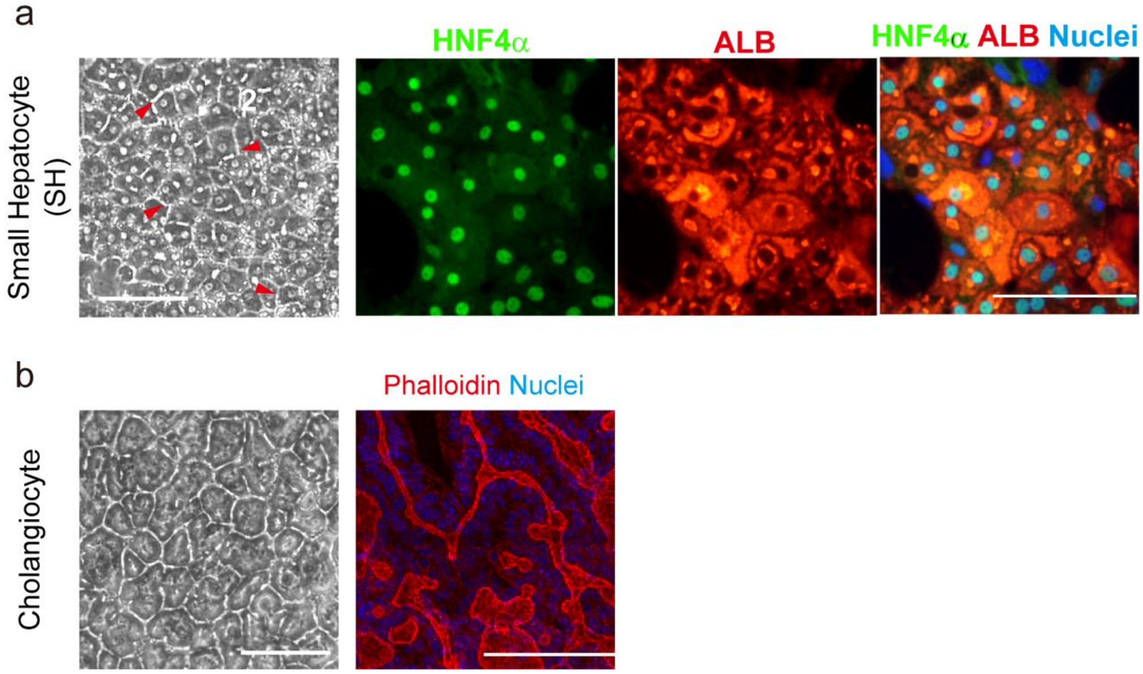

Supplementary Fig. 1. Morphogenesis of SHs and cholangiocytes in culture.

a. **SHs differentiate to acquire cellular characteristics similar to mature hepatocytes (MHs).** SHs show cellular morphologies similar to MHs, including round nuclei and dense cytoplasm in the presence of OSM and Matrigel (phase-contrast image in the left panel). SHs form bile canaliculi (red arrowheads), but they are not organized into a network. SHs become expressing HNF4α (green) and ALB (red). SHs were cultured on a gelatin-coated dish for five days, then kept in the presence of OSM for one day, and overlaid with Matrigel. The immunostaining with anti- HNF4α (green), anti-ALB (red) antibodies, and Hoechst 33342 (blue) was repeated three times independently. Three fields were examined in each sample and representative images are shown in this figure. Bars represent 100 μm.

b. **Cholangiocytes form tubular networks in sandwich culture.** Cholangiocytes proliferate on type I collagen gel and form tubular structures after the overlay of collagen gel (left panel). The luminal network is visualized by phalloidin (right panel). The staining with phalloidin (red) and hoechst33342 (blue) was repeated three times independently. Three fields were examined in each sample and representative images are shown in this figure. Bars represent 100 μm.

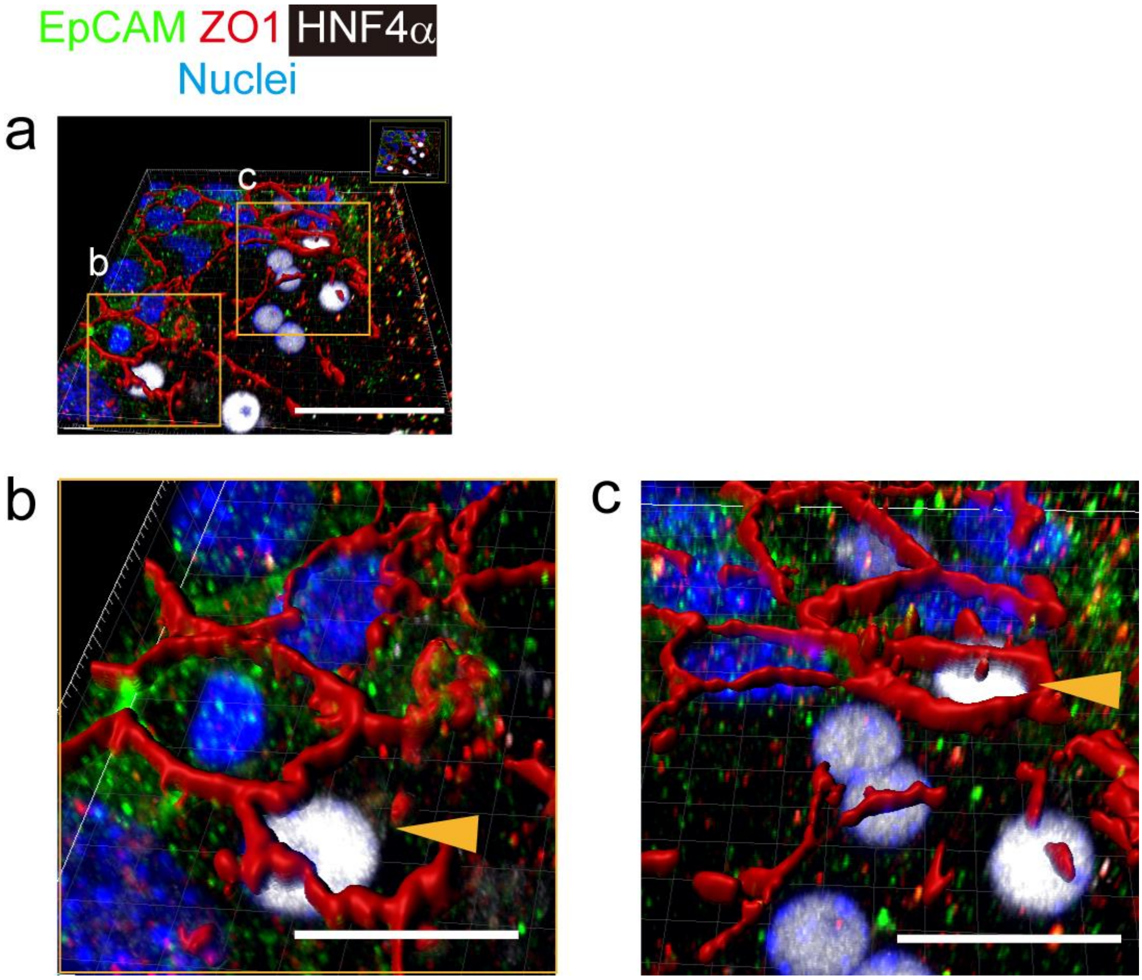

Supplementary Fig. 2. SHs and cholangiocytes quickly form intercellular junctions.

HNF4α^{+} hepatocytes (arrowheads in panels b & c) form tight junctions with EpCAM^{+} cholangiocytes. Boxes in panel a are enlarged in panels b and c. Tight junctions are visualized with ZO1 staining. A surface model was generated on Imaris. The immunostaining with anti-EpCAM (green), anti-ZO1 (red), anti-HNF4α (white) antibodies, and Hoechst 33342 (blue) was repeated twice independently. Three fields were examined in each sample. The surface models for the representative image were constructed on Imaris and are shown in this figure. Bars in panel a, and panels b & c represent 50 and 20 μm, respectively.

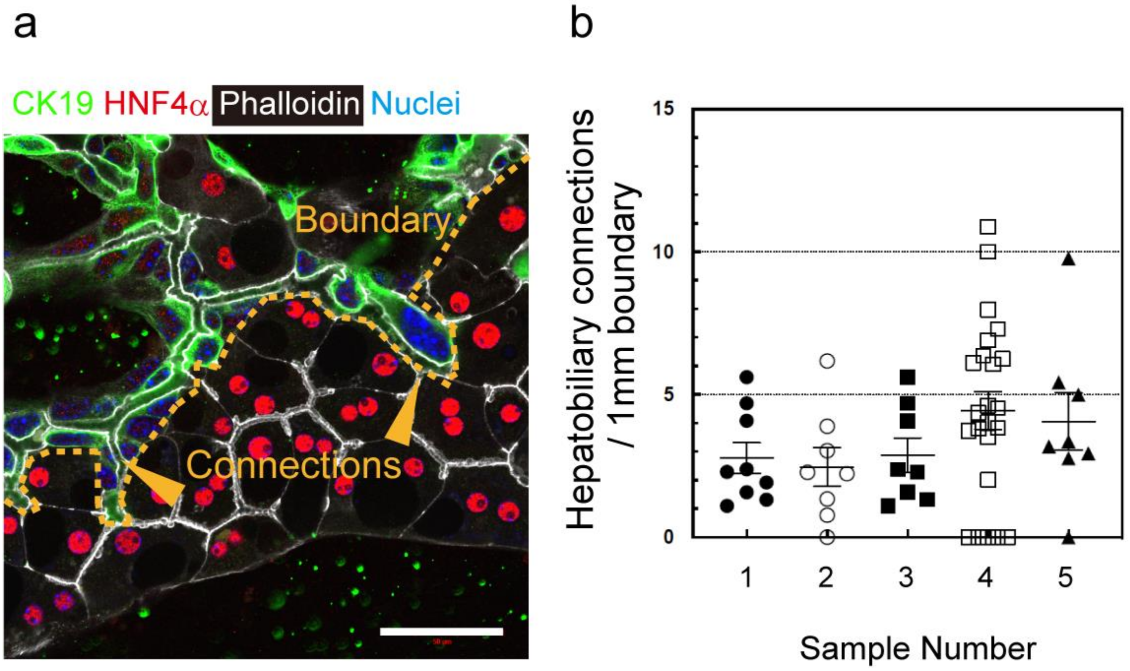

Supplementary Fig. 3. Quantification of the number of hepatobiliary connections.

a. HBTOs were stained with anti-HNF4α, anti-CK19 and phalloidin. The boundary between hepatocytes and cholangiocytes was marked by a broken line to measure its length on Olympus cellSens software. The phalloidin^{+} luminal structures connecting the hepatocyte clusters and biliary tubules were counted (**arrowheads**). The immunostaining with anti-CK19 antibody (green), anti-HNF4α antibody (red), phalloidin (white), and Hoechst 33342 (blue) was repeated five times independently and the representative image is shown in this figure. The bar represents 50 μm.

b. The number of connections per 1 mm boundary was calculated. More than eight areas were selected from five independent culture wells for examining the length of the boundary and the connections (n=5). Mean ± SEM of each culture sample is shown in the graph. The average values of five culture samples were used for determining the number of hepatobiliary connections shown in the text.

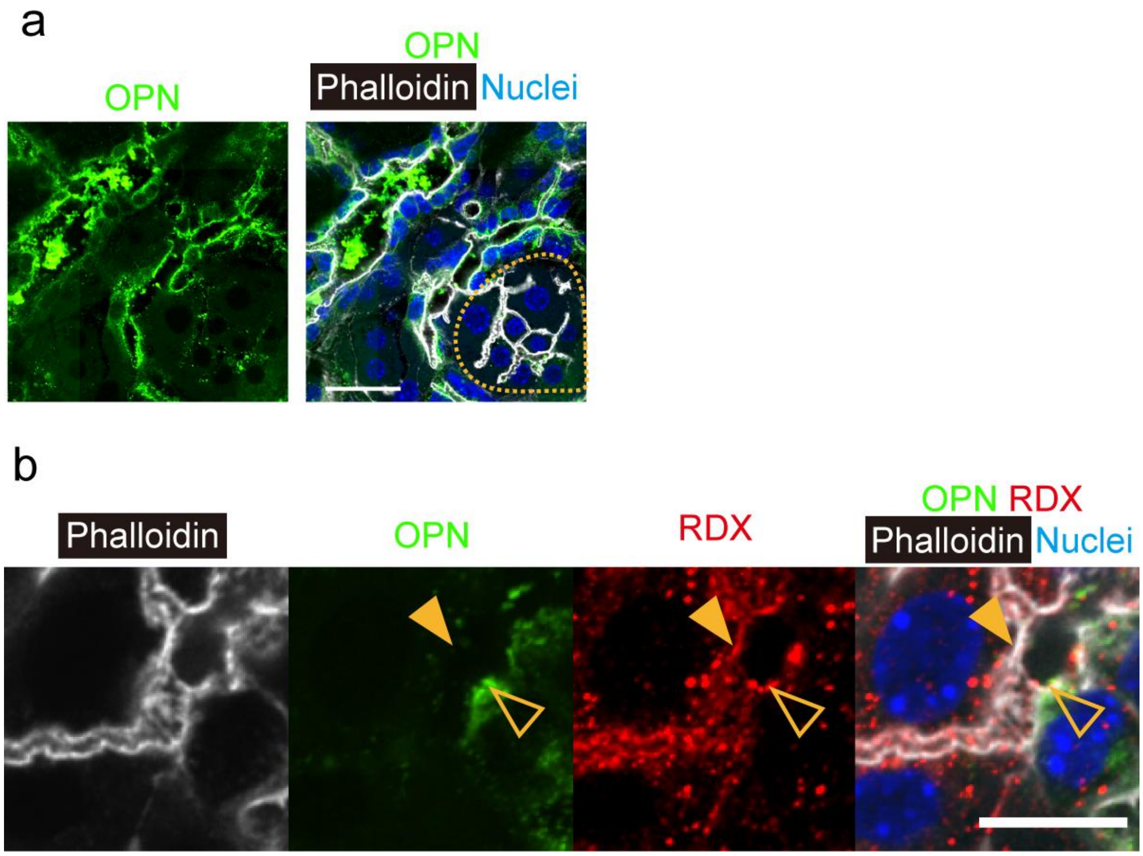

Supplementary Fig. 4. Expression of OPN and RDX in HBTO.

a. Cholangiocytes in HBTO express OPN. OPN (green) is expressed in cholangiocytes but not in hepatocytes (surrounded by a yellow dotted line). The immunostaining with anti-OPN antibody (green), anti-phalloidin (white), and Hoechst 33342 (blue) was repeated twice independently. Three fields were examined in each sample and the representative images are shown in this figure. The bar represents 50 μm.

b. Expression of OPN and RDX in the hepatobiliary junction. The apical membrane of an OPN^+ cholangiocyte (open arrowhead) and that of a RDX^+ hepatocyte (closed arrowhead) surround the luminal structure at the hepatobiliary junction. The immunostaining with phalloidin (white), anti-OPN antibody (green), anti-RDX antibody (red), and Hoechst 33342 (blue) was repeated twice independently. Three fields were examined in each sample and the representative images are shown in this figure. The bar represents 20 μm.

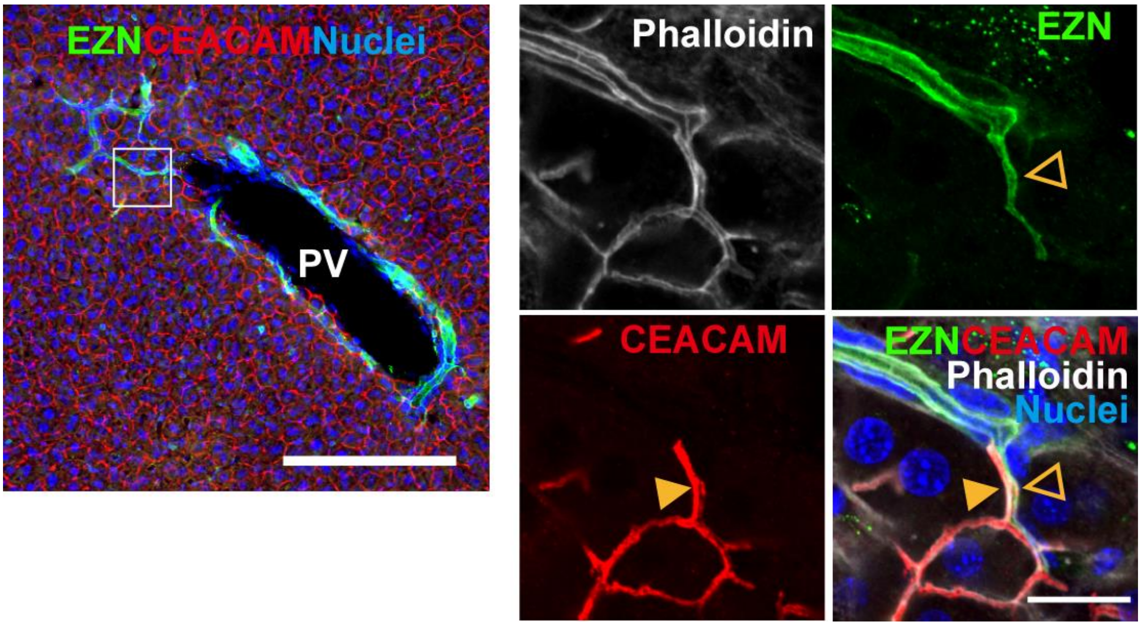

Supplementary Fig. 5. Hepatobiliary connection *in vivo*

Bile canalicular membrane and the lumen of bile ducts are positive for CEACAM and EZN, respectively (left panel). At the connection between the bile canaliculi and the bile duct, $CEACAM^{+}EZN^{-}$ hepatocytes (closed arrowheads) and $CEACAM^{-}EZN^{+}$ cholangiocytes (open arrowheads) surround the lumen. The immunostaining with phalloidin (white), anti-EZN antibody (green), anti-CEACAM antibody (red), and Hoechst 33342 (blue) was repeated on sections prepared from two different mice. Three fields were examined on each section and the representative images are shown in this figure. The box in the left is enlarged in the right four panels. Bars represent 200 and 50 μm, respectively.

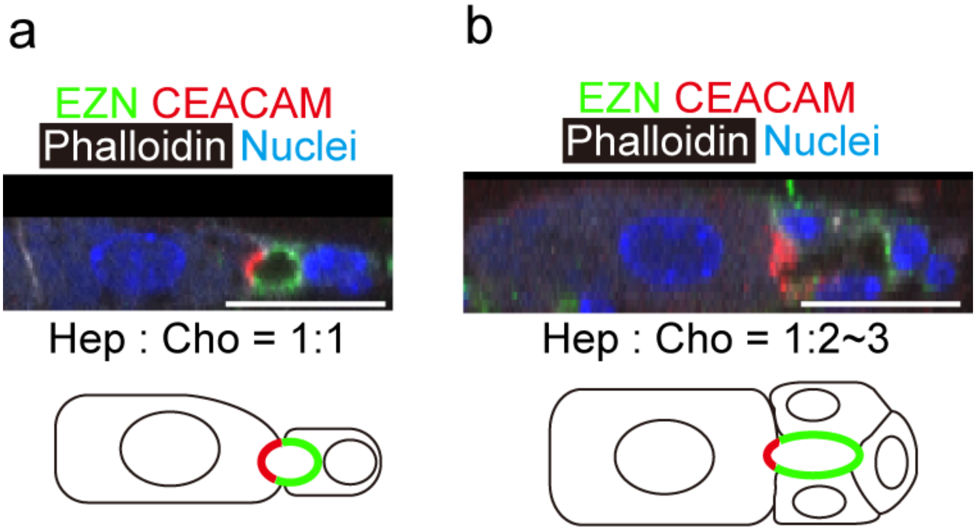

c

Type	Hep:Cho = 1:1	Hep:Cho = 1:2~3
Number of connections	31/52	21/52
Ratio (%)	60	40

Supplementary Fig. 6. 3D analysis for the hepatobiliary connection

CEACAM^{+} apical domain of hepatocyte and EZN^{+} apical domain of cholangiocyte surround the luminal structure at the hepatobiliary connection. The connection consists of one hepatocyte and one cholangiocyte (a) or one hepatocyte and two or three cholangiocytes (b). X-Y images were collected every 0.5 μm along the Z-axis on a confocal microscope. Three samples cultured independently were stained with anti-EZN antibody (green), anti-CEACAM antibody (red), phalloidin (white), and Hoechst 33342 (blue). In addition, three samples cultured independently were stained with anti-CK19, anti-ALB, phalloidin, and Hoechst 33342. In total, six samples were used for 3D analysis. Six to ten areas containing hepatobiliary connections were identified in each sample. In total, 52 connections were examined and hepatocytes and cholangiocytes involved in the connection were counted (table c). Bars represent 20 μm.

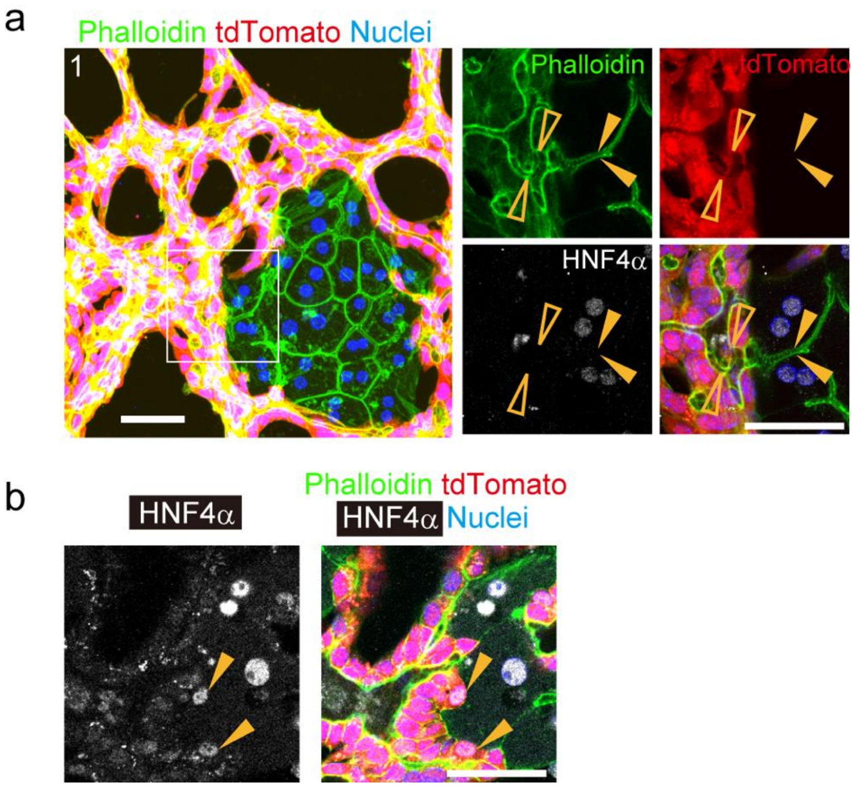

Supplementary Fig. 7. Co-culture of tdTomato$^+$ cholangiocytes and wild type SHs.

a. **tdTomato$^+$ cholangiocytes keep their lineage to form a continuous luminal network with hepatocytes.** The luminal network among tdTomato$^-$HNF4α$^+$ hepatocytes (**closed arrowheads**) is connected to that of Tomato$^+$ biliary structure (**open arrowheads**) at the boundary. The luminal network is recognized by F-actin bundles visualized with AlexaFluor488-conjugated phalloidin. The immunostaining with phalloidin (green), anti-HNF4α antibody (white), and Hoechst 33342 (blue) was repeated three times independently. Two fields were examined in each sample and the representative images are shown in this figure. The box in the left panel is enlarged in the right four panels. Bars represent 50 μm.

b. **Some tdTomato$^+$ cholangiocytes express HNF4α but maintain cholangiocyte morphology.** Some tdTomato$^+$ cholangiocytes express HNF4α but its expression level was lower than that in hepatocytes (arrowheads). The HNF4α^{low} cells showed cellular morphology similar to neighboring cholangiocytes. The culture and immunostaining were repeated three times and two different areas in each sample were examined to detect tdTomato$^+$HNF4α^{low} cells. One or two HNF4α^{low} cholangiocytes were detected in two of six fields, whereas HNF4α^{low} cholangiocytes were not observed in other fields. The bar

represents 50 μm.

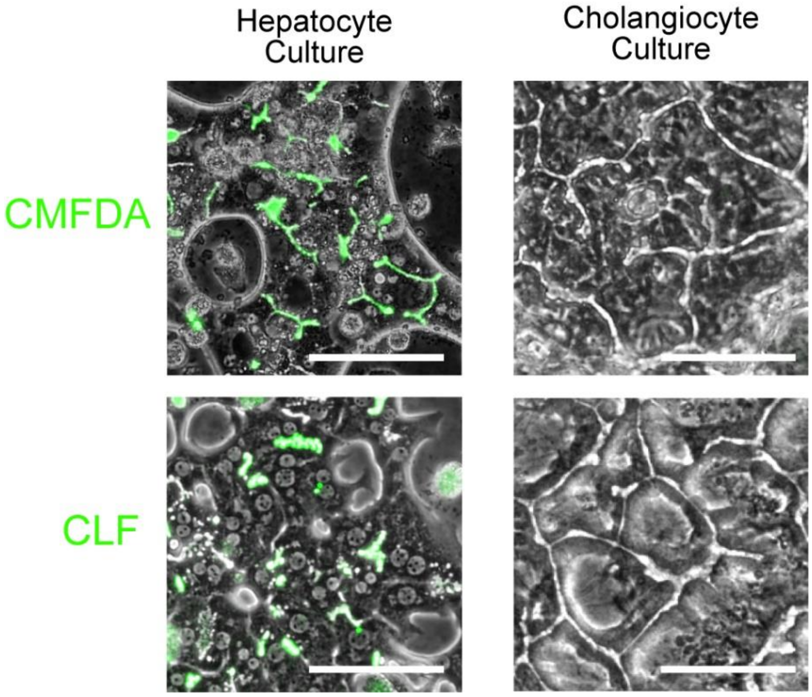

Supplementary Fig. 8. Hepatocytes but not cholangiocytes take up CMFDA and CLF. Hepatocytes take up chloromethyl fluorescein diacetate (CMFDA), metabolize it, and secrete fluorescein into BCs. They also absorb cholyl-lysine fluorescein (CLF) and secrete it into BC (upper and lower left panels). In contrast, cholangiocytes take up neither CMFDA nor CLF, and, therefore, fluorescein is not observed in the luminal space (upper and lower right panels). SHs and cholangiocytes were cultured on type I collagen gel and overlaid with Col-MG. Two weeks after Col-MG overlay, they were incubated in the medium with CMFDA for 30 min or with CLF for two hours. Images were taken on a fluorescence microscope after five times wash. Experiments were repeated twice independently. Three fields were examined in each sample and the representative images are shown in this figure. Bars represent 100 μm.

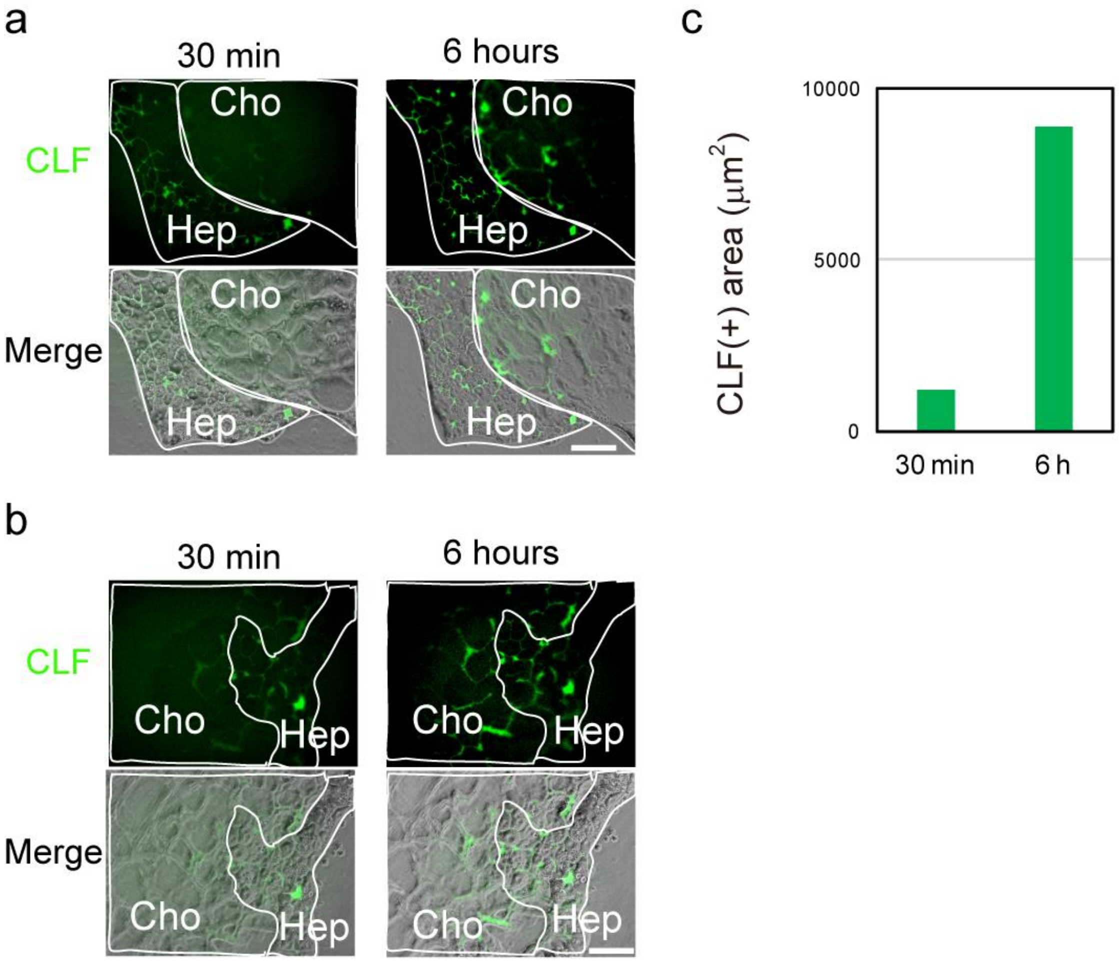

Supplementary Fig. 9. Quantificaiton of transport of CLF in HBTOs.

CLF is accumulated in BCs in hepatocyte clusters (Hep) during first 30 min of incubation. By six hours, CLF is transported to biliary tubules (Cho). The transport of CLF in two independent cultures is shown in this figure (panels a and b). The fluorescence intensity in the biliary tubules (Cho) was qunatified at 30 min and at 6 hours using imageJ. The average values are shown in the graph (c). Panel a is the same culture shown in Fig. 3b. Bars represent 100 μm.

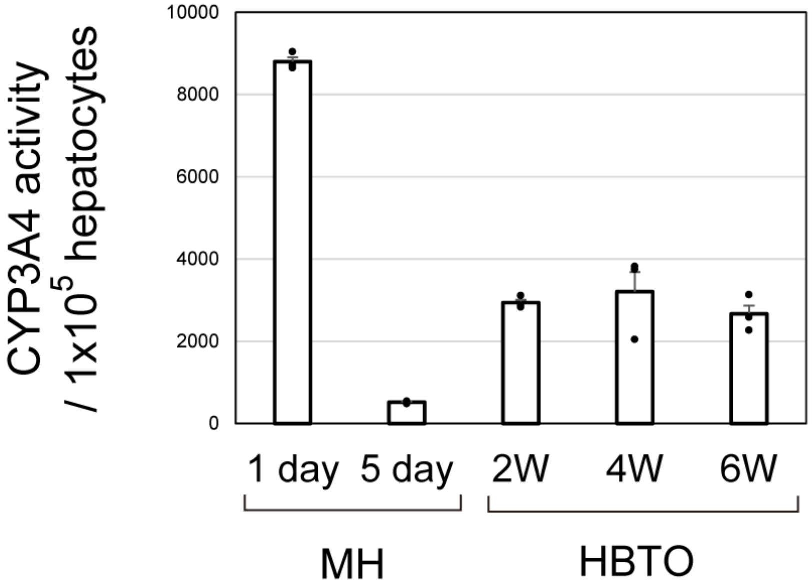

Supplementary Fig. 10. Primary MHs lose Cyp activity in culture.

CYP3A4-like activity in MHs declined to less than one-twentieth during five days of culture. Although CYP3A4-like activity in HBTO was about one-fourth of that in MHs at one day, it was maintained for a month. Primary MHs (1×10^5 cells) were cultured in 24-well plates coated with type I collagen. CYP3A4-like activity was measured at one and five days after plating. HBTO were formed initially from SHs (5×10^4 cells) and cholangiocytes (5×10^4 cells) in 24 well plates. CYP3A4-like activities were measured in three different wells of MHs and HBTO culture. Average values of CYP3A4-like activity per 1×10^5 hepatocytes are shown in the graph. Error bars represent SEMs.

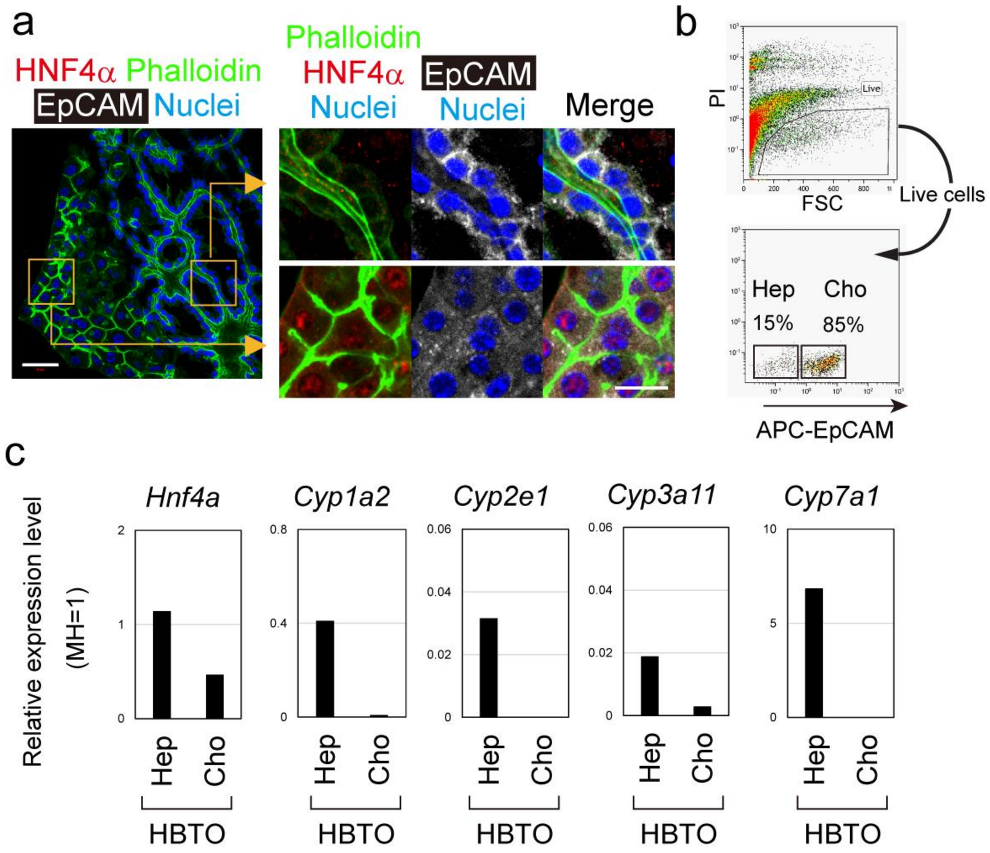

Supplementary Fig. 11. Expression of hepatocyte markers in HBTO

a. Cholangiocytes but not hepatocytes express EpCAM in HBTO. The immunostaining with anti-HNF4α (red), anti-EpCAM (white) antibody, phalloidin (green), and Hoechst 33342 (blue) was repeated twice independently. Three fields were examined in each sample and the representative images are shown in this figure. Boxes in the left panel are enlarged in right panels. Upper and lower three images show cholangiocytes and hepatocytes, respectively. Bars represent 50 and 20 μm, respectively.

b. Separation of cholangiocytes and hepatocytes derived from HBTO. HBTO was digested with Liberase TM to liberate cells, and hepatocytes and cholangiocytes were isolated as EpCAM(-) and EpCAM(+) cells, respectively. The FACS plot is representative of two independent cell isolations.

c. *Cyps* are specifically expressed in hepatocytes in HBTO. Expression of hepatocyte markers was examined using qPCR. Their expression levels are presented as relative values against those in MHs cultured for one day. Cell isolation and PCR analyses were repeated twice, independently. The average value of two cell isolations are shown in the graphs.

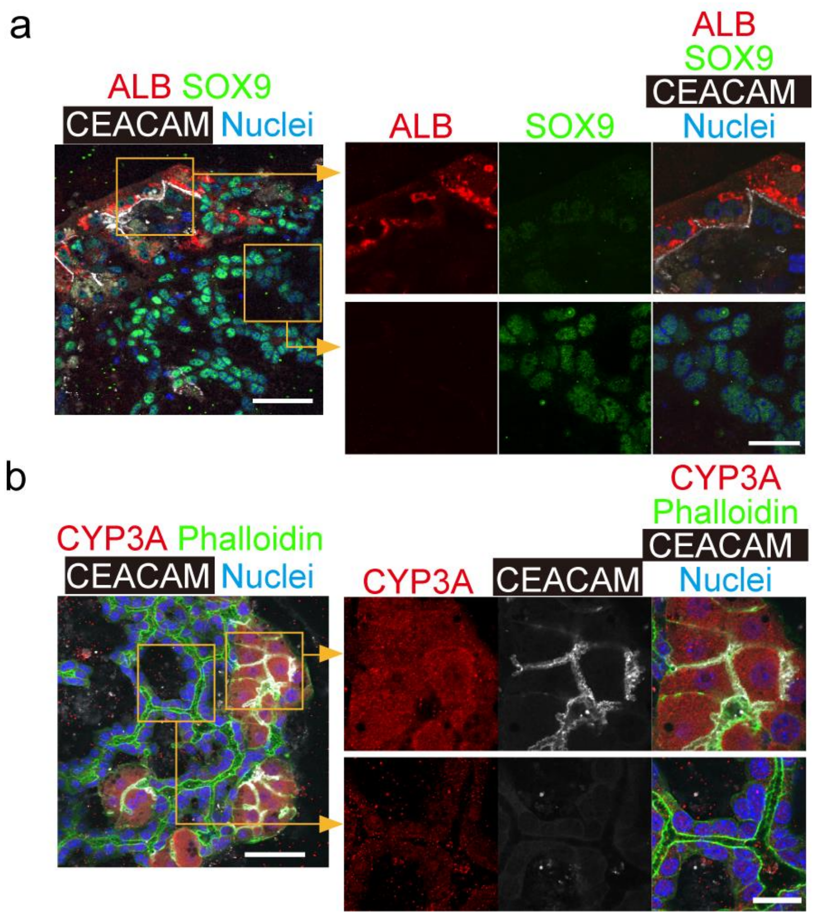

Supplementary Fig. 12. Expression of ALB and CYP3A in HBTO.

a. **ALB is expressed in CEACAM$^+$ hepatocytes but not in SOX9$^+$ cholangiocytes.** The immunostaining with anti-ALB (red), anti-SOX9 (green), anti-CEACAM (white) antibody, and Hoechst 33342 (blue) was repeated twice independently. Three fields were examined in each sample and representative images are shown in this figure. Boxes in the left panel are enlarged in the right panels. Upper and lower three panels show hepatocytes and cholangiocytes, respectively. Bars in the left and right images represent 50 and 20 μm, respectively.

b. **CYP3A is expressed in CEACAM$^+$ hepatocytes.** HBTOs were stained with anti-human CYP3A4 (Abcam, Cambridge, UK). The antibody is reported to be cross-reactive to mouse protein. Mouse CYP3A11, whose mRNA is expressed in HBTOs, has 69% homology to human CYP3A4, suggesting mouse CYP3A11 is detected with this antibody. The immunostaining with anti-CYP3A antibody (red), phalloidin (green), anti-CEACAM antibody (white), and Hoechst 33342 (blue) was repeated twice independently. Three fields were examined in each sample and representative images are shown in this figure. Boxes in the left panel are enlarged in the right panels. Upper and lower three panels show hepatocytes and cholangiocytes, respectively. Bars in left and right panels represent 50 and 20 μm, respectively.

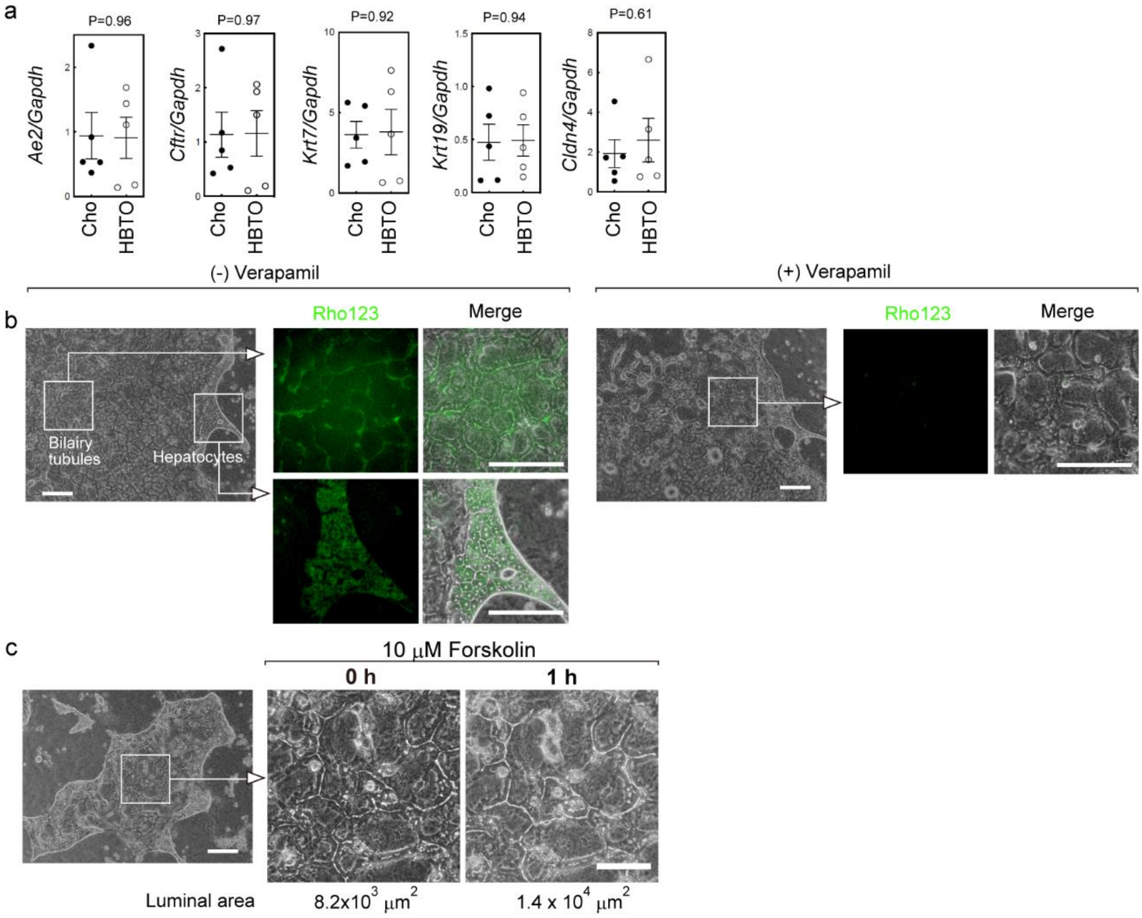

Supplementary Fig. 13. Cholangiocytes are functional in HBTO.

a. **Cholangiocytes in the organoid express cholangiocyte marker genes.** Expression levels of *Ae2*, *Cftr*, *Krt7*, *Krt19*, and *Cldn4* are similar between Cho and HBTO, suggesting that cholangiocytes in HBTO maintain cellular characteristics as differentiated cholangiocytes. Cholangiocytes (Cho) were maintained in sandwich culture for four weeks, under the same culture conditions as HBTO. cDNA was prepared from five wells for both Cho and HBTO and used for qPCR analysis (n=5). Error bars represent SEM. Unpaired two-tailed *t*-tests were performed using Microsoft Excel.

b. **Cholangiocytes take up Rho 123 depending on Mdr1 activity.** HBTO was incubated in the presence of Rho123 without ((-)verapamil) or with verapamil((+)verapamil). Rho123 is absorbed by cholangiocytes and accumulated in the biliary tubules. It is absorbed by hepatocytes, but not accumulated in the BC. The accumulation of Rho123 in the biliary tubules is blocked by verapamil, a MDR1 inhibitor. Images are representative of two independent experiments. Boxes are enlarged in square panels. Bars represent 200 μm.

c. **Cholangiocytes expand the luminal space responding to Forskolin.** The luminal space of the biliary tissue is expanded about twofold in the presence of Forskolin. HBTO was incubated in the presence of Forskolin for one hour. Images are representative of two independent experiments. The luminal spaces in the area shown in right panels were quantified using ImageJ. The box in left panel is enlarged in the middle one, and an image of the same area at one hour after treatment is shown in the right. Bars represent 200μm.

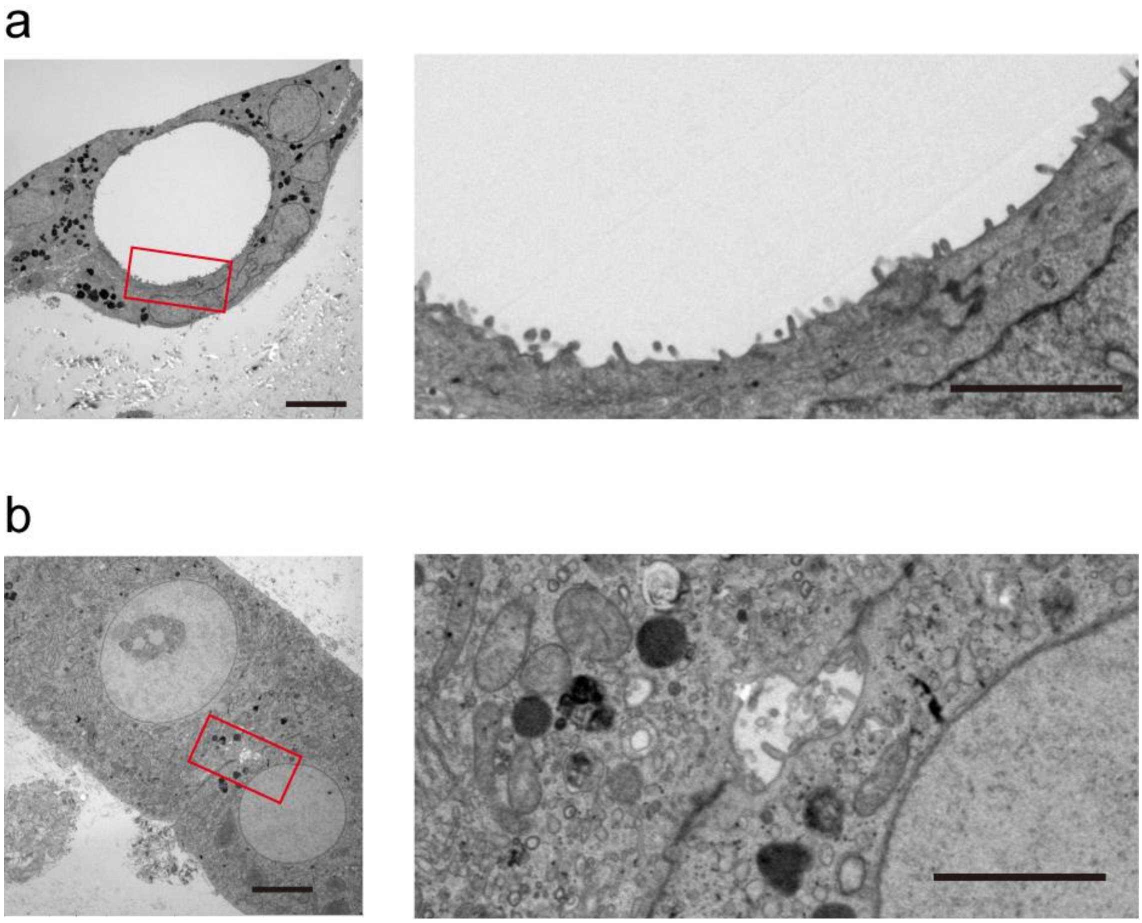

Supplementary Fig. 14. Transmission electron microscopic (TEM) analysis for cellular morphologies of cholangiocytes and hepatocytes in HBTOs.

a. **Cholangiocyte structure.** Cholangiocytes have microvilli on their apical surface. The red box in the left panel is enlarged in the right. Bars in left and right panels represent 10 and 5 μm, respectively.

b. **Hepatocyte structure.** Hepatocytes form bile canaliculi between neighboring cells. The red box int the left panel is enlarged in the right. Bars in left and right panels represent 10 and 5 μm, respectively. TEM samples prepared from two independent culture samples and images were taken for five different areas per sample. Representative images are shown in this figure.

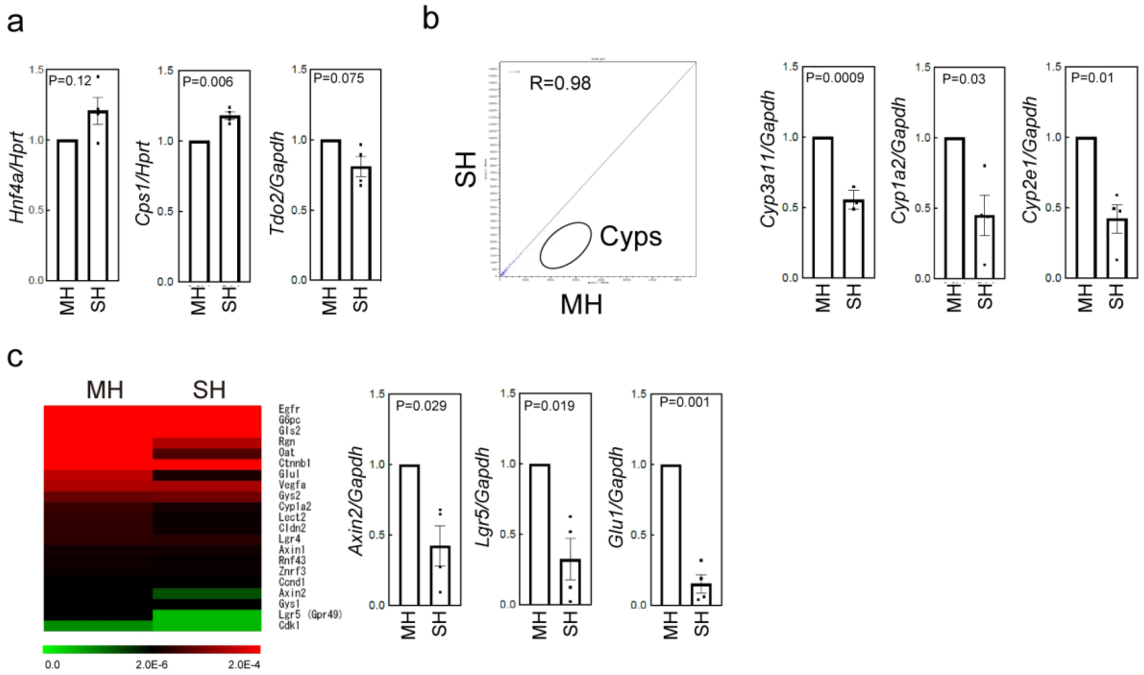

Supplementary Fig. 15. Comparison of gene expression of SHs with that of MHs.

a. **SHs expression hepatocyte markers.** *Hnf4a* is expressed more and *Tdo2* is expressed less in SHs as compared with those in MHs. *Cps1* is expressed significantly more in SHs. SHs and MHs were isolated from the same mouse for four times, independently. These four sets of SHs and MHs were used to examine expression of *Hnf4a*, *Tdo2*, and *Cps1* (n=4). Error bars represent SEM.

b. Cyps are expressed significantly less in SHs than those in MHs. RNA sequence data suggests that expression of Cyps is less in SHs than in MHs (graph in left). qPCR analyses further demonstrate that expression of *Cyp3a11*, *Cyp1a2*, and *Cyp2e1* are significantly less in SHs than in MHs. Four sets of SHs and MHs were used to examine expression of *Cyp3a11*, *Cyp1a2*, and *Cyp2e1* (n=4). Error bars represent SEM.

c. WNT target genes are expressed significantly less in SHs than in MHs. RNA sequence data suggests expression of WNT target genes is less in SHs than in MHs (Heatmap). qPCR analyses further demonstrate that expression of *Axin2*, *Lgr5*, and *Glu1* are significantly less in SHs than in MHs. Four sets of SHs and MHs were used to examine expression of *Axin2*, *Lgr5*, and *Glu1* (n=4). Error bars represent SEM. Paired two-tailed *t*-tests were performed for all qPCR data shown in this figure using Microsoft Excel.

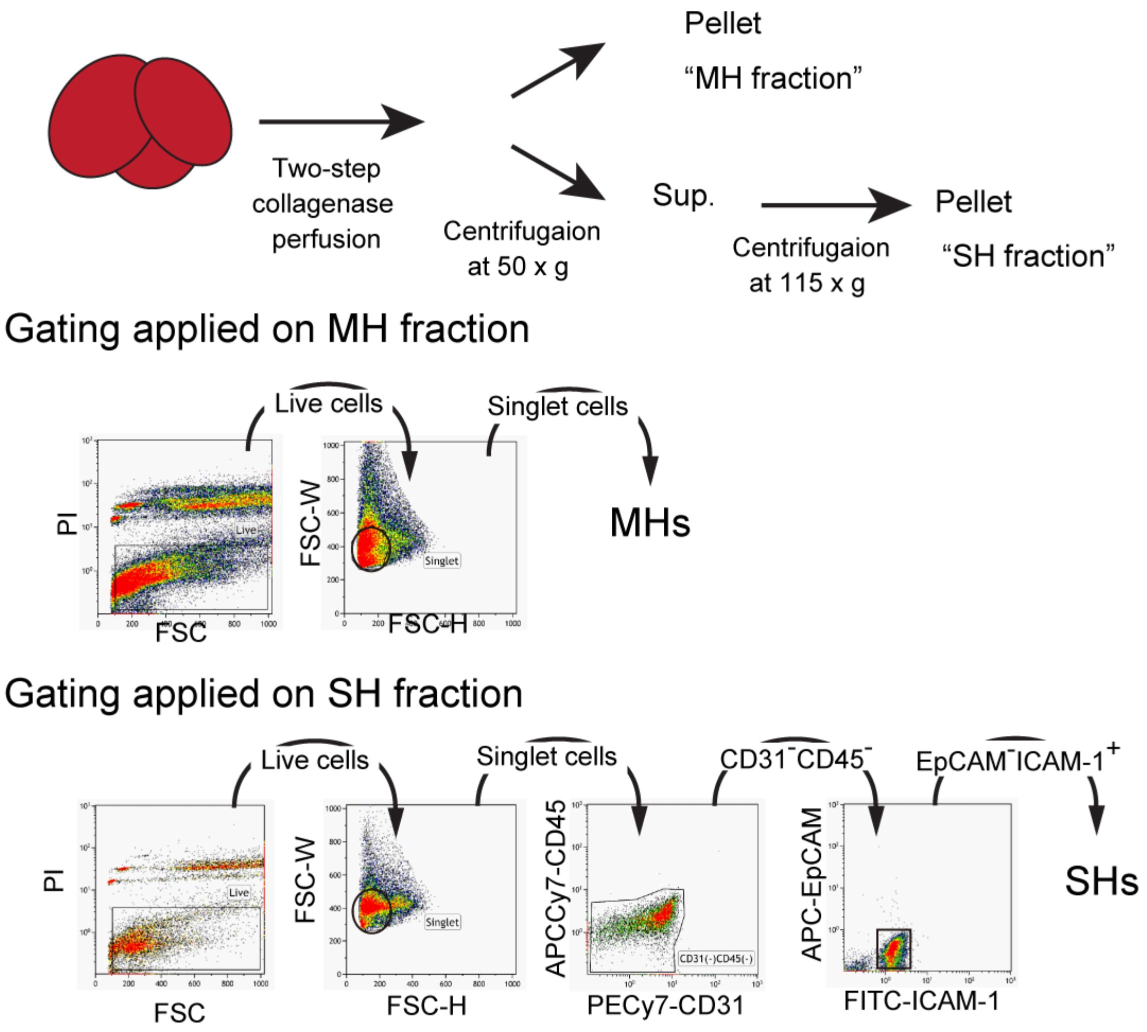

Supplementary Fig. 16. Isolation and FACS analysis of MHs and SHs.

MHs and SHs were enriched by centrifugation at differnet gravity. Live singlet cells were selected to analyze ECAD expression in MHs. Live singlet cells were further separated to CD31$^-$CD45$^-$ and then EpCAM$^-$ICAM-1$^+$ cells to analyze ECAD expression in SHs. FACS plots shown in this figure are representative of three independnet experiments.

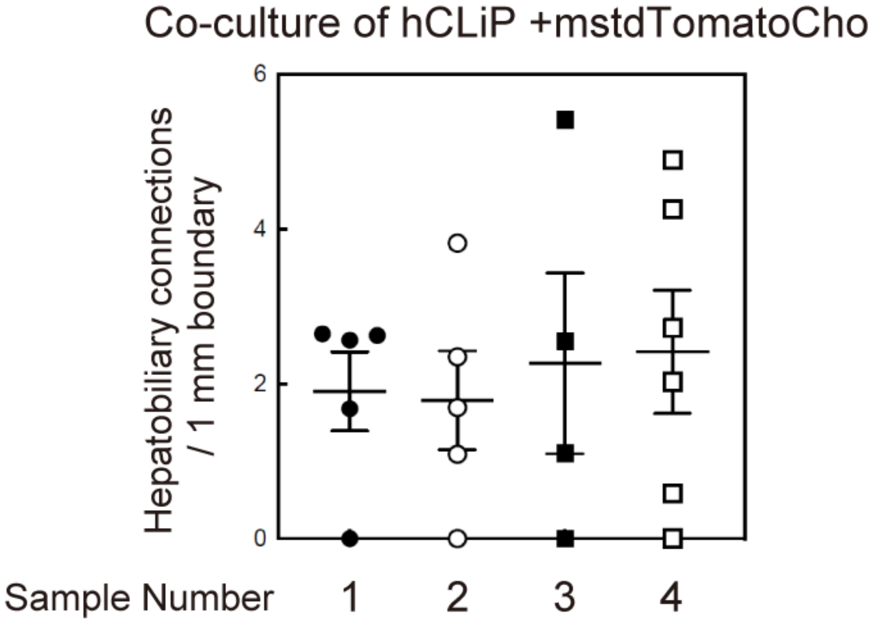

Supplementary Fig. 17. Quantification of the number of hepatobiliary connection in HBTOs consisting of human reprogramming hepatocytes (hCLiP) and mouse tdTomato^{+} cholangiocytes (mstdTomatoCho).

Coculture of hCLiP with mstdTomatoCho was repeated four times, independently (n=4). HBTOs were stained with anti-HNF4α and phalloidin. The length of the boundary between hepatocytes and cholangiocytes was measured using Olympus cellSens software. The phalloidin^{+} luminal structures connecting the hepatocyte clusters and biliary tubules were counted. The number of connections per 1 mm boundary was calculated and the Mean ± SEM was plotted. More than four different areas per well were selected, and used to measure the length of the boundary and count the connections. The average values of the examined areas in each well were calculated, and used to determine the number of hepatobiliary connections, as shown in the text.

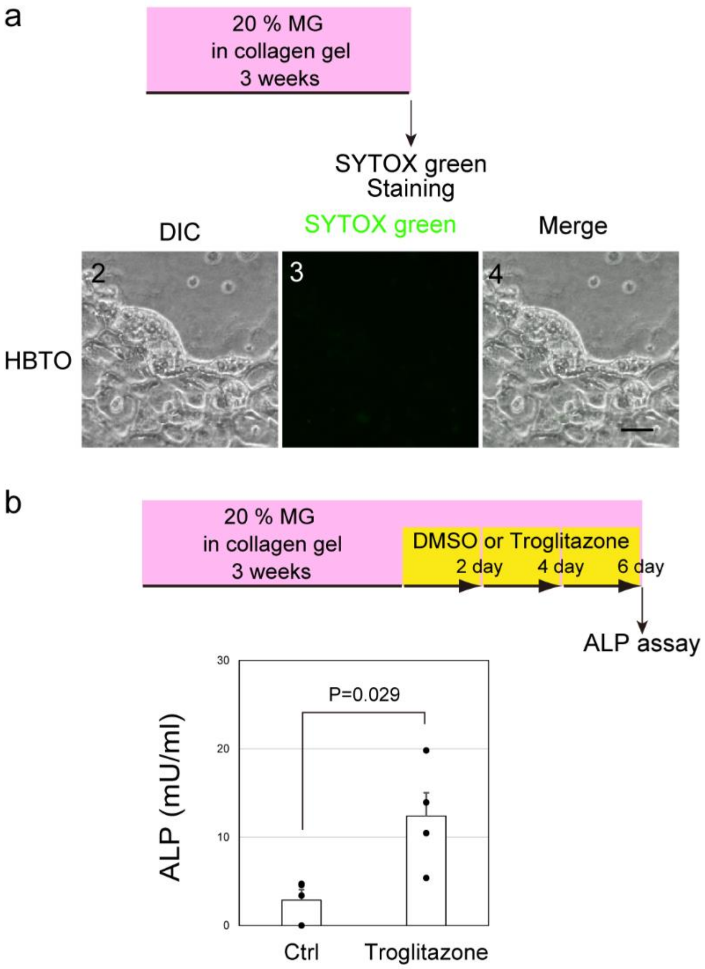

Supplementary Fig. 18. Cell survival and death in HBTOs.

a. **HBTOs do not contain dead hepatocytes and cholangiocytes.** Hepatocytes and cholangiocytes constructing HBTOs are alive at three weeks after Col-MG overlay. HBTOs were incubated in medium containing SYTOX green for 20 min and examined under a fluorescence microscope. No cells showed positive signal. The culture and SYTOX staining were repeated twice independently. Three fields were examined in each sample and the representative images are shown in this figure. The bar represents 100 μm.

b. **Hepatocytes in HBTOs release ALP in the presence of troglitazone.** ALP is released from damaged hepatocytes in HBTOs treated with 200 μM troglitazone for six days. The medium was replaced with fresh medium with or without troglitazone every two days. Four wells per treatment were used for assay and the average values with SEMs are shown in this graph. Unpaired two-tailed *t*-tests were performed using Microsoft Excel.

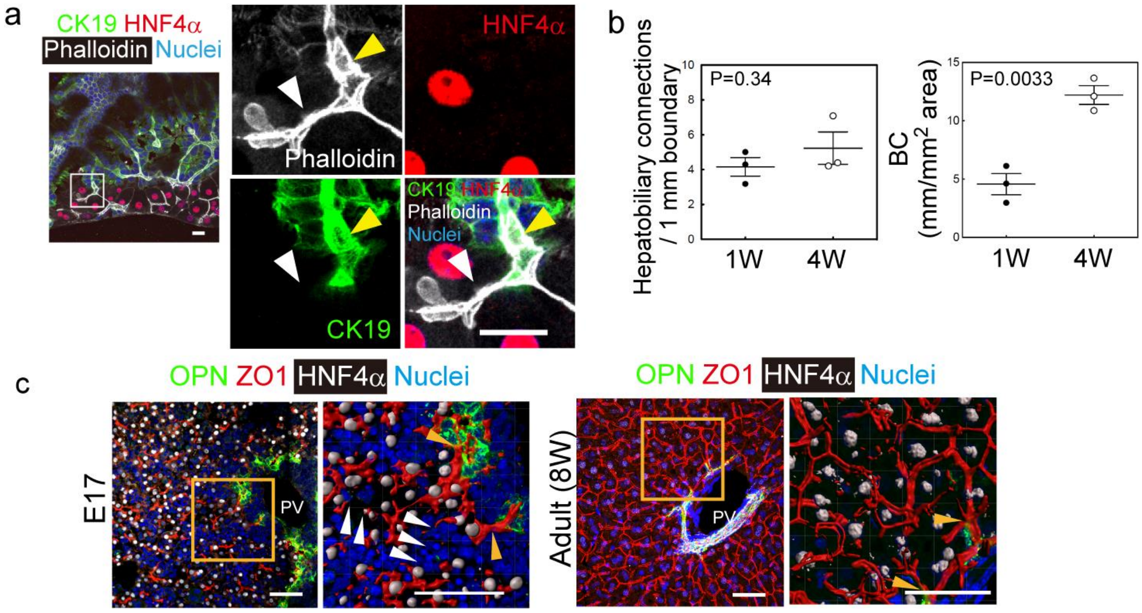

Supplementary Fig. 19. Hepatocytes and biliary epithelial cells form the junctional structure at the onset of liver epithelial morphogenesis.

a. **The hepatobiliary connection in co-culture.** The apical luminal structure (white) in HNF4α$^+$ hepatic (white arrowhead) and that in CK19$^+$ biliary tubule (yellow arrowhead) is already connected one week after MG-Col overlay. Box in the left panel is enlarged in right four panels. The immunostaining with anti-CK19 antibody (green), and anti-HNF4α antibody (red), phalloidin (white), and Hoechst 33342 (blue) was repeated three times independently. Three fields were examined in each sample and the representative images are shown in this figure. Bars represent 40 μm.

b. **The number of hepatobiliary connection and the length of the bile canaliculi in HBTOs at one and four weeks after MG-Col overlay.** Hepatobiliary connections are evident at one week after MG-Col overlay and their number does not significantly increase by four weeks. On the other hand, BCs are formed at one week after MG-Col overlay, and further extended by four weeks. Four to six areas were selected in three independent culture samples stained with anti-EZN, anti-CEACAM, and phalloidin. The length of hepatobiliary boundary and that of the BCs were measured using Olympus cellSens software. The phalloidin$^+$ luminal structures connecting the hepatocyte clusters and biliary tubules were counted. The average values of each sample were plotted in this graph. Error bars represent SEM. Unpaired two-tailed *t*-tests were performed using Microsoft Excel.

c. **Hepatobiliary connections in fetal and adult livers.** BCs are still discontinuous at E17 (white arrowheads), whereas hepatobiliary connections are already established around the portal vein at this stage (yellow arrowheads). On the other hand, the apical luminal network is continuous among hepatic cords and IHBDs in the adult liver (yellow arrowheads show hepatobiliary connections). Confocal images collected from 20 μm thickness and projection images were reconstituted on Zeiss Zen software. The boxes were cropped and surface models were constructed on Imaris. The immunostaining with anti-OPN (green), anti-ZO1 (red), anti-HNF4α (white) antibodies, and Hoechst 33342 (blue) was repeated on thick sections prepared from two different mice both at E17 and at adult. Two fields were examined on each sections and the representative images were used to construct the surface model. Bars represent 50 μm.

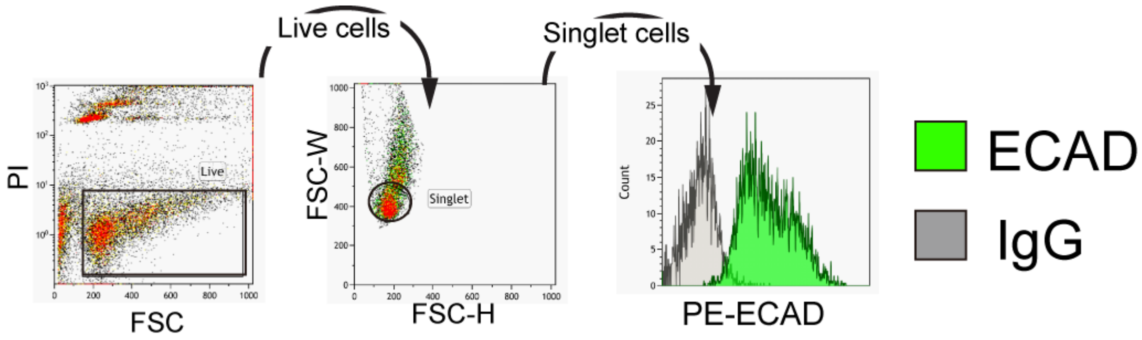

Supplementary Fig. 20. ECAD expression in cholangiocytes.

Cholangiocytes were cultured for five days on type I collagen gel. Cholangiocytes were isolated from culture by digesting collagen gel with Liberase TM and then examined for expression of ECAD on FACS. The FACS plots are representative of two independent analyses.

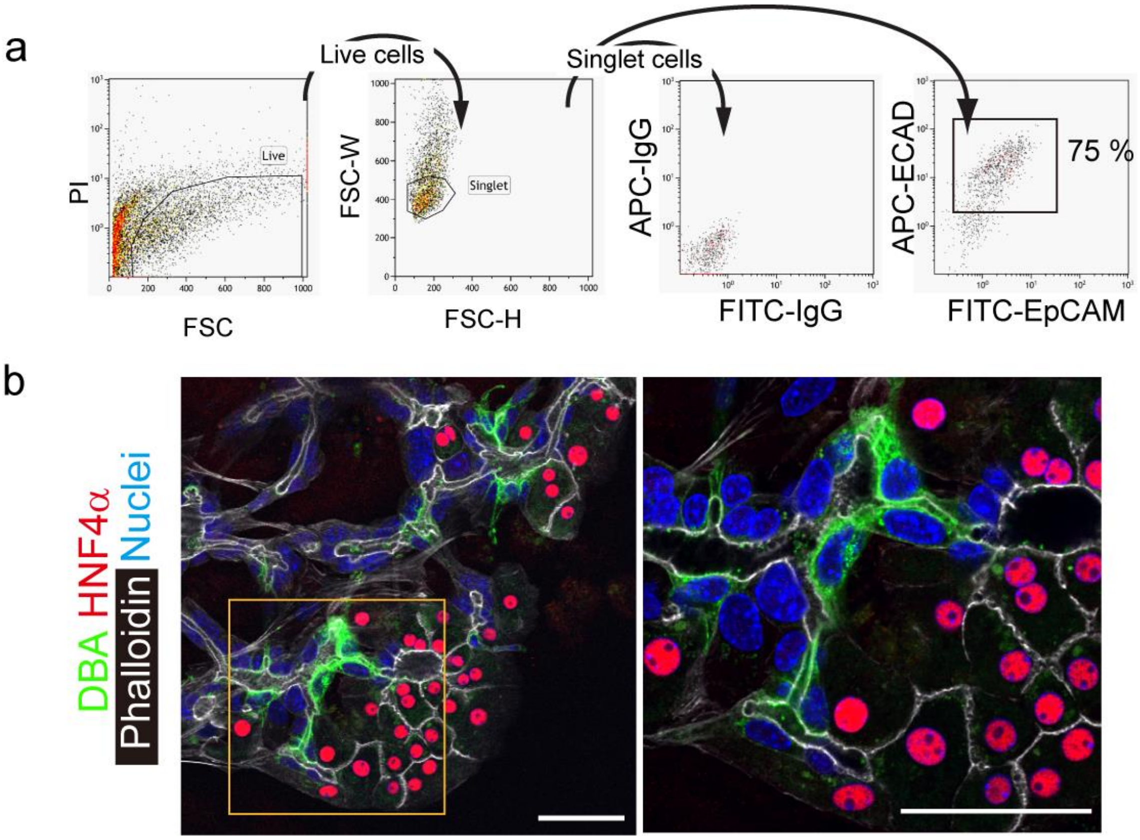

Supplementary Fig. 21. Coculture of pancreatic duct cells and SHs.

After five days of culture on type I collagen gel, pancreatic duct cells were EpCAM^{+}ECAD^{+}. One week after Col-MG overlay, pancreatic ducts and hepatocyte clusters occasionally formed organotypic structures. The phalloidin^{+} luminal structure is continuous between the BC in the HNF4α^{+} hepatocyte cluster (red) and the dolichos biflorus agglutinin (DBA)$^{+}$ pancreatic duct structure (green). FACS analysis were repeated twice and the representative FACS plots are shown in this figure. The immunostaining with FITC conjugated-DBA (green), anti-HNF4α antibody (red), phalloidin (white), and Hoechst 33342 (blue) was repeated twice independently. Three fields were examined in each sample and the representative images are shown in this figure. The box in the left panel is enlarged in the right one. Bars represent 50 μm.
