# Supplementary material for: Generation of functional liver organoids on combining hepatocytes and cholangiocytes with hepatobiliary connections ex vivo
Source: Nat Commun. 2021 Jun 7;12:3390. doi: 10.1038/s41467-021-23575-1 (PMC8185093; doi:10.1038/s41467-021-23575-1)
Supplement: Supplementary file 2 — Reporting Summary [file 41467_2021_23575_MOESM2_ESM.pdf]

nature research

Corresponding author(s): Naoki Tanimizu

Last updated by author(s): May 5, 2021

Reporting Summary

Nature Research wishes to improve the reproducibility of the work that we publish. This form provides structure for consistency and transparency in reporting. For further information on Nature Research policies, see our Editorial Policies and the Editorial Policy Checklist.

Statistics

For all statistical analyses, confirm that the following items are present in the figure legend, table legend, main text, or Methods section.

n/a	Confirmed	
☐	☒	The exact sample size (*n*) for each experimental group/condition, given as a discrete number and unit of measurement
☐	☒	A statement on whether measurements were taken from distinct samples or whether the same sample was measured repeatedly
☐	☒	The statistical test(s) used AND whether they are one- or two-sided<br>*Only common tests should be described solely by name; describe more complex techniques in the Methods section.*
☒	☐	A description of all covariates tested
☐	☒	A description of any assumptions or corrections, such as tests of normality and adjustment for multiple comparisons
☐	☒	A full description of the statistical parameters including central tendency (e.g. means) or other basic estimates (e.g. regression coefficient) AND variation (e.g. standard deviation) or associated estimates of uncertainty (e.g. confidence intervals)
☐	☒	For null hypothesis testing, the test statistic (e.g. *F*, *t*, *r*) with confidence intervals, effect sizes, degrees of freedom and *P* value noted<br>*Give P values as exact values whenever suitable.*
☒	☐	For Bayesian analysis, information on the choice of priors and Markov chain Monte Carlo settings
☒	☐	For hierarchical and complex designs, identification of the appropriate level for tests and full reporting of outcomes
☒	☐	Estimates of effect sizes (e.g. Cohen's *d*, Pearson's *r*), indicating how they were calculated

Our web collection on statistics for biologists contains articles on many of the points above.

Software and code

Policy information about availability of computer code

Data collection	Zeiss ZEN, Olympus cellSens
Data analysis	Microsoft excel, Karuza ver1.1, Imaris ver9, ImageJ ver1.48, GraphPad Prism ver5

For manuscripts utilizing custom algorithms or software that are central to the research but not yet described in published literature, software must be made available to editors and reviewers. We strongly encourage code deposition in a community repository (e.g. GitHub). See the Nature Research guidelines for submitting code & software for further information.

Data

Policy information about availability of data

All manuscripts must include a data availability statement. This statement should provide the following information, where applicable:

- Accession codes, unique identifiers, or web links for publicly available datasets
- A list of figures that have associated raw data
- A description of any restrictions on data availability

RNA sequence data is deposited in the Gene Expression Omnibus with the accession code GSE166283 with hyperlink https://www.ncbi.nlm.nih.gov/geo/query/acc.cgi?acc=GSE166283. Data points shown in figures are provided as Source Data. All other relevant data are available from the corresponding author.

Field-specific reporting

Please select the one below that is the best fit for your research. If you are not sure, read the appropriate sections before making your selection.

- [x] Life sciences
- [ ] Behavioural & social sciences
- [ ] Ecological, evolutionary & environmental sciences

For a reference copy of the document with all sections, see nature.com/documents/nr-reporting-summary-flat.pdf

Life sciences study design

All studies must disclose on these points even when the disclosure is negative.

Sample size	We did not perform any statistical analysis to determine sample size. Hepatobiliary connections were basically observed in all cultures and immunostaining shown in the manuscript gave the similar results in each experiment. Two or three independent experiments including cell isolation, culture, and immunostaining are performed to secure reproducibility. For quantitative analyses examining hepatocyte functions and gene expression, we repeated experiments at least three times, independently. For quantitative analyses examining organoid structures, we also repeated culture and immunostaining three times independently. Since we expected relatively high variability, we selected at least three different fields, randomly, of each culture well to examine organoid structures including the length of the boundary between hepatocytes and cholangiocytes, and hepatobiliary connection. More details about sample size are provided in Methods and figure legends.
Data exclusions	No data excluded from analysis.
Replication	Organoids in 3 different wells were used to measure enzyme activities. Duplicate for each sample was generated for CYP assays and qPCR analyses. Other experiments, three to five samples cultured independently were used for immunostainning and analyses. All the replicates were successfully provided data points.
Randomization	We did not perform randomization. In the current work, we used primary cholangiocytes isolated from the one mouse strain, C57BL6. When comparing SHs with MHs, we isolated them from the same mice. We consistently used the same culture conditions. Therefore, there are not parameters for randomization.
Blinding	We performed blind test for Fig. 5e. We randomly labeled immunostaining images of HBTO containing ECAD(-) MHs or ECAD(+) MHs with numbers. Without pre-knowledge the nature of hepatocytes in organoid, the number of hepatobiliary connection was counted. For Supplementary Fig. 19b, since the length of BC are obviously different in any field between HBTOs at 1W and those at 4W, we did not perform blind test. For other experiments comparing two experimental groups, data points were obtained by instruments automatically, and therefore, we did not perform blind test.

Reporting for specific materials, systems and methods

We require information from authors about some types of materials, experimental systems and methods used in many studies. Here, indicate whether each material, system or method listed is relevant to your study. If you are not sure if a list item applies to your research, read the appropriate section before selecting a response.

Materials & experimental systems

n/a	Involved in the study	
[ ]	[x]	Antibodies
[x]	[ ]	Eukaryotic cell lines
[x]	[ ]	Palaeontology and archaeology
[ ]	[x]	Animals and other organisms
[x]	[ ]	Human research participants
[x]	[ ]	Clinical data
[x]	[ ]	Dual use research of concern

Methods

n/a	Involved in the study	
[x]	[ ]	ChIP-seq
[ ]	[x]	Flow cytometry
[x]	[ ]	MRI-based neuroimaging

Antibodies

Antibodies used	We provide required information in Supplementary Table 2 and 3
Validation	We validated specificity of antibodies performing staining on mouse liver sections. Cholangiocytes specifically express CK19, SOX9, OPN, and EZN, whereas hepatocytes exclusively express CEACAM, HNF4a, and RDX. Therefore, using frozen sections prepared from healthy adult mice, we can check the specificity of those antibodies whether each signal is detected exclusive in cholangiocytes forming bile ducts or hepatocytes in the liver parenchyma.<br>FACS analyses, we always prepared a negative control where cells were incubated with unimmunized rat immunoglobulin to verify the specific reaction of a primary antibody used for examination of a surface marker and for cell isolation.

Animals and other organisms

Policy information about studies involving animals; ARRIVE guidelines recommended for reporting animal research

Laboratory animals	We used male/female C57BL6 mice. Ages were between 8 and 12 week-old. They were maintained in environment with constant light/dark cycle (12 hours), ambient temperature (23±2 degree), and humidity (40%). This information is provided in Methods.
Wild animals	No wild animals were used in the study.
Field-collected samples	No field collected samples were used in the study.
Ethics oversight	All animal experiments were approved by the Sapporo Medical University Institutional Animal Care (Experimental code: 18-094) and Use Committee and were conducted according to institutional guidelines for ethical animal use.

Note that full information on the approval of the study protocol must also be provided in the manuscript.

Flow Cytometry

Plots

Confirm that:

- [x] The axis labels state the marker and fluorochrome used (e.g. CD4-FITC).
- [x] The axis scales are clearly visible. Include numbers along axes only for bottom left plot of group (a 'group' is an analysis of identical markers).
- [x] All plots are contour plots with outliers or pseudocolor plots.
- [x] A numerical value for number of cells or percentage (with statistics) is provided.

Methodology

Sample preparation	Primary cholangiocytes and hepatocytes were isolated from healthy adult mice by a two-step collagenase perfusion. SHs were collected from fraction after elimination of MHs by centrifugation at 50 x g. Live cells were enriched by Percoll density gradient centrifugation. Cholangiocytes were further purified as EpCAM(+) cells by MACS. ECAD(-) and ECAM(+) MHs were isolated by FACS. hCLiPs derived from frozen human hepatocytes were provided by Prof. Ochiya's group were maintained on collagen coated dish and trypsinized before using co-culture with cholangiocytes. For qPCR analysis, SHs were further enriched in CD31(-)CD45(-)EpCAM(-)ICAM-1(+) cells by FACS.
Instrument	ABI-Prism7500, Zeiss LSM780, Olympus FV3000, BD FACSAriaII, Beckman coulter Moflo, Miltenyi MACS, Biotek 800TS absorbance reader
Software	Zeiss ZEN, Olympus cellSens, Microsoft excel, Karuza ver1.1, Imaris ver9, ImageJ ver1.48, GraphPad Prism ver5
Cell population abundance	Cholangiocyte fraction purified by MACS shows 50% purity judged by EpCAM expression. In the following culture on collagen gel, the purity becomes over 90% when SHs are plated. SHs fraction contains hematopoietic cells and, therefore, the purity is about 50%. However, no hematopoietic cells attached to collagen gel and only SHs remained in culture 2 days after plating.
Gating strategy	PI(-) live cells were used to select singlet cells on a FSC-H and FSC-W plot. Singlet cells were used to analyze expression of ECAD and EpCAM, and cell isolation. For isolating cells from HBTOs, we isolated EpCAM(-) hepatocytes and EpCAM(+) cholangiocytes from PI(-) live cells.

- [x] Tick this box to confirm that a figure exemplifying the gating strategy is provided in the Supplementary Information.
